# Miraculin Can Contribute to a Reduction in Inflammatory Biomarkers and Cachexia in Malnourished Patients with Cancer and Taste Disorders

**DOI:** 10.3390/ph18050622

**Published:** 2025-04-25

**Authors:** Ana Isabel Álvarez-Mercado, Bricia López-Plaza, Julio Plaza-Diaz, Lucía Arcos-Castellanos, Francisco Javier Ruiz-Ojeda, Marco Brandimonte-Hernández, Jaime Feliú-Batlle, Thomas Hummel, Samara Palma-Milla, Ángel Gil

**Affiliations:** 1 Department of Pharmacology, University of Granada, 18071 Granada, Spain; 2 Instituto de Investigación Biosanitaria IBS.GRANADA, Complejo Hospitalario Universitario de Granada, 18014 Granada, Spainagil@ugr.es (Á.G.); 3 Centre of Biomedical Research, Institute of Nutrition and Food Technology “José Mataix”, University of Granada, Avda. del Conocimiento s/n, Armilla, 18016 Granada, Spain; 4 Food, Nutrition and Health Platform, Hospital La Paz Institute for Health Research (IdiPAZ), 28046 Madrid, Spain; 5 Medicine Department, Faculty of Medicine, Complutense University of Madrid, Plaza de Ramón y Cajal, s/n, 28040 Madrid, Spain; 6 Department of Biochemistry and Molecular Biology II, University of Granada, 18071 Granada, Spain; 7 School of Health Sciences, Universidad Internacional de La Rioja, Avenida de la Paz, 137, 26006 Logrono, Spain; 8 CIBEROBN (CIBER Physiopathology of Obesity and Nutrition), Instituto de Salud Carlos III, 28029 Madrid, Spain; 9 RU Adipocytes and Metabolism, Helmholtz Diabetes Center at Helmholtz Munich, German Research Center for Environmental Health GmbH, 85764 Neuherberg, Germany; 10 Oncology Department, Hospital La Paz Institute for Health Research—IdiPAZ, Hospital Universitario La Paz, 28029 Madrid, Spain; 11 CIBERONC (CIBER Cancer), Instituto de Salud Carlos III, 28029 Madrid, Spain; 12 Medicine Department, Faculty of Medicine, Autonomous University of Madrid, Arzobispo Morcillo 4, 28029 Madrid, Spain; 13 Smell & Taste Clinic, Department of Otorhinolaryngology, Technische Universität Dresden, Fetscherstraße 74, 01307 Dresden, Germany; 14 Nutrition Department, Hospital University La Paz, 28046 Madrid, Spain

**Keywords:** cachexia, cancer, DMB, miraculin, dysgeusia, inflammation, neoplasia, nutritional status, taste disorders

## Abstract

**Background:** In 2022, there were an estimated 20 million new cancer cases and 9.7 million deaths. The number of new cancer cases is expected to rise to over 35 million by 2050, marking a 75% increase from 2022 levels. Twenty to eighty-six percent of cancer patients suffer from taste disorders (TD), which are associated with an increased risk of malnutrition. Cachectic syndrome is linked to the presence and growth of tumors and leads to systemic inflammation. *Synsepalum dulcificum* is a plant whose berries contain miraculin, a glycoprotein that transforms sour tastes into sweet and can ameliorate TD. **Objectives:** To evaluate the effect of the regular intake of dried miracle berries (DMBs), a novel food containing miraculin, on biomarkers of inflammation and cachexia in malnourished patients with cancer and TD receiving systemic antineoplastic therapy. **Methods:** we conducted a triple-blind, randomized, placebo-controlled pilot clinical trial. Thirty-one patients with cancer of various etiologies who received chemotherapy were enrolled in this pilot study and divided into three groups. The first group received a tablet containing 150 mg of DMB (standard dose), the high-dose group received a tablet of 300 mg of DMB, and the third group received a tablet with 300 mg of the placebo for three months before each main meal. The plasma levels of several molecules associated with inflammation and cancer cachexia were measured using the X-MAP Luminex multiplexing platform. **Results**: We found decreased plasma levels of IFN-γ in the standard-dose group. In addition, our results suggest a downtrend of IL-1β levels in the three groups after three months of intervention (*p* = 0.093). Moreover, the three groups showed a reduction in tumor-derived molecule proteolysis-inducing factor/dermcidin (*p* = 0.021). It is important to highlight the positive correlation between IL-6 and IL-10 in the standard group, which suggests a better balance between proinflammatory and anti-inflammatory cytokines. Regardless of DMB consumption, soluble TNF receptor type II tended to decrease with treatment in patients who responded well to the antineoplastic treatment (*p* = 0.011). We did not find significant correlations between cytokines and sensory variables or dietary and nutritional status. **Conclusions:** Our results suggest that the regular consumption of a standard dose of DMB along with a systemic antineoplastic treatment could contribute to reducing inflammation and cachexia biomarkers in malnourished patients with cancer exhibiting TD. In this sense, nutritional support is crucial in the treatment of cancer cachexia. In our view, it should be considered a coadjuvant of therapeutics. Future studies on the molecular signaling pathways and specific mechanisms of action of bioactive compounds within food supplements, such as miraculin, will allow them to be used to target pathogenic mechanisms of cancer cachexia and malnutrition: NCT05486260.

## 1. Introduction

According to the WHO, in 2022, approximately 20 million new cancer cases were diagnosed worldwide, of which 9.7 million patients died. Projections for 2050 indicate that new cancer cases are expected to rise to over 35 million, marking a 75% increase from 2022 levels [1,2].

The most common treatments for cancer are surgery, chemotherapy, and radiation. Other treatment options include targeted therapy, immunotherapy, laser therapy, or hormonal therapy [2]. Chemotherapy and radiotherapy can cause taste and smell disorders by altering the structure of the pores of the palate with consequent thinning of the epithelium of the papilla [3], affecting taste nerves and salivary glands, and destroying taste cells, including the eradication of proliferative progenitors [4]. Those disorders alter the pleasure produced by taste and smell through the formation of conditioned aversions. In this regard, the term dysgeusia refers to quantitative or qualitative taste dysfunction, including taste distortions with bitter, metallic, salty, or unpleasant tastes [5,6]. As a result, since food is perceived as unpleasant, a lower intake of food can contribute to weight loss and malnutrition in patients with cancer. Indeed, foods consumed can cause nausea, which negatively impacts the quality of life and nutritional status [5,7]. In severe cases, malnutrition can progress to cachexia, which is also a common condition in many patients with cancer [8]; this complex metabolic disorder is characterized by a pronounced loss of muscle and fat mass, systemic inflammation, weakness, and fatigue [9].

Cachexia can also develop in patients suffering from chronic diseases, such as chronic heart failure, chronic obstructive pulmonary disease, chronic kidney disease, acquired immunodeficiency syndrome (AIDS), rheumatoid arthritis, Alzheimer’s disease and dementia, or sepsis and other severe infections [10].

Certain cancers also induce the systemic reprogramming of the host’s energy metabolism, leading to glucose, lipid, and protein turnover alterations. These metabolic imbalances are promoted by tumor-secreted and tumor-induced factors that promote cachexia [9].

Chronic systemic inflammation is also a critical component of cachexia. Inflammatory cytokines, particularly tumor necrosis factor-alpha (TNF-α), interleukin (IL)-6, and interferon-γ (IFN-γ), are major drivers of many symptoms of the disease and are thought to be responsible for the metabolic changes associated with tissue loss in cancer wasting [11,12]. In contrast, the cytokine IL-15, which is essential for the development, proliferation, and activation of immune cells, can also enhance the antitumor activity of immune cells and has been shown to possess significant antitumor potential [13]. IL-10, a multifunctional cytokine with multiple properties, has been extensively studied in various immunology and cancer biology fields. IL-10 is a pleiotropic cytokine that promotes cytotoxicity, although high levels of IL-10 can inhibit antitumor responses [14]. On the other hand, when present systemically, ciliary neurotrophic factor (CNTF) is involved in the induction of cachexia, induces the catabolism of stored fat, skeletal muscle protein, and liver glycogen, and decreases the circulating concentrations of several intermediary metabolites [5]. Moreover, tumor-derived molecules such as proteolysis-inducing factor/dermcidin (PIF) have also been proposed as mediators of cancer cachexia. PIF induces protein degradation in skeletal muscle via the ubiquitin-proteasome proteolytic pathway. Notably, cancer cachexia is a strong independent cause of mortality in cancer patients [9], and those suffering from cancer cachexia are generally less tolerant of chemotherapies and radiotherapies, limiting their treatment options [15].

Unfortunately, cancer patients are involved in a vicious cycle in which illness provokes decreased food intake, malabsorption, and/or increased loss of nutrients, leading to an increase in susceptibility to chemotherapy-induced toxicity. Ultimately, these complications, together with reduced mobility, fatigue, poor response to therapy, and other complications, can further compromise nutritional status, and the cachectic state becomes self-perpetuating [16]. Overall, products developed to prevent or alleviate cachexia should meet both the nutritional and sensory needs of cancer patients, promote the enjoyment of food, counteract malnutrition, stabilize weight, and improve quality of life [7]. In this sense, *Synsepalum dulcificum* (Daniell) is a plant whose berries contain miraculin, a glycoprotein that transforms sour flavors into sweet ones and limits bitter and metallic off-flavors, making food more palatable. In December 2021, the European Commission authorized the dried fruits of *S. dulcificum* (also known as DMB), which naturally contain the taste-modifying protein miraculin, as a novel food [17]. Recently, our group reported that regular consumption of DMB before each main meal for three months improved taste acuity and salty taste, leading to greater dietary intake (energy intake, fat quantity, and quality), and ameliorated nutritional status (fat-free mass) and erythrocyte polyunsaturated fatty acid (PUFA) status [17]. Indeed, an improvement in nutritional status could have a positive impact on inflammatory and cachexia states. Considering those mentioned above, in this study, we hypothesized that miraculin intake, by improving the perception of bitter and metallic taste in patients with cancer, could increase their appetite for food, thereby improving their nutritional status and, consequently, their condition. To test our hypothesis and evaluate the effect of regular consumption of DMB on inflammatory and cachexia states, we evaluated changes in several biomarkers of those conditions in moderately malnourished patients with cancer suffering from taste disorders (TDs) and receiving systemic antineoplastic therapy.

The analysis of any pilot study should be primarily descriptive due to the small number of patients included. In this sense, the preliminary effects allowed us to determine the degree to which the intervention succeeded in producing favorable results in achieving the goals set. Moreover, due to the small number of participants allowed, pilot studies do not often yield statistically significant results. Nevertheless, pilot studies provide a guideline for Phase III RCTs for intervention development, where the efficacy and effectiveness of the intervention are evaluated [18,19].

## 2. Results

We used a linear mixed model of covariance to examine the differences between the placebo, 150 mg of DMB, and 300 mg of DMB over the 3-month treatment period. To evaluate whether the antineoplastic treatment influenced the DMB effect, we used cancer treatment success as a covariate and expressed it as a dichotomous variable (0 = no success; 1 = success); we also included radiotherapy and chemotherapy as additional covariates. We did not observe changes in the DMB effect relative to the antineoplastic treatment.

Firstly, we analyzed changes in the circulating levels of several biomarkers of inflammation and cachexia in patients who regularly consumed DMB (standard dose and high dose) or placebo before and after three months of intervention (Table 1). The three groups showed decreased plasma levels of IL-4 throughout the intervention.

INF-γ decreased significantly only in the standard dose group, and IL-1β decreased in the placebo group. Nevertheless, our results suggest a downtrend of IL-1β levels in the three groups after three months of intervention (*p* = 0.093). In addition, while the circulating levels of sTNFR-I decreased in the high-dose group over time, we observed a significant reduction (*p* = 0.011) in sTNFR-II in the three groups, especially in the standard-dose group. The same pattern was found for the tumor-derived molecule PIF (*p* = 0.021, overall after 3 months).

**Figure 1:**
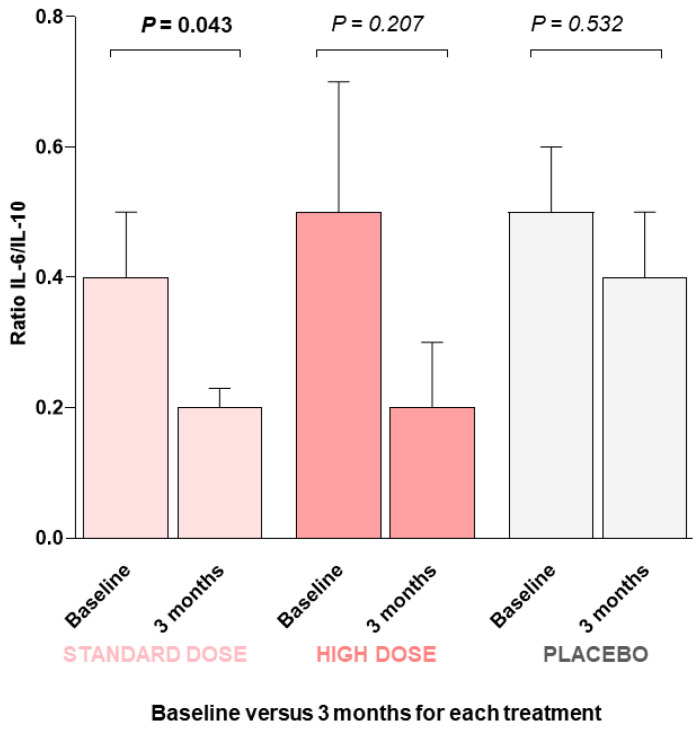
IL-6-to-IL-10 ratio at the beginning of this study (baseline) and after three months of intervention with DMB or placebo. A GLM-ANCOVA statistical test was performed. Values are the means ± SEM. *p* value < 0.05 was considered to indicate statistical significance.

Then, we determined the IL-6/IL-10 ratio to establish the balance between pro- and anti-inflammatory cytokines. At the beginning of this study, all three groups had similar values for this parameter (0.4 ± 0.08, standard dose; 0.5 ± 0.2, high dose; 0.5 ± 0.1, placebo group). However, after 3 months of intervention, the groups that ingested the standard dose of DMB showed the greatest reduction in the ratio (0.2 ± 0.03, *p* = 0.043, standard dose; 0.2 ± 0.1, *p* = 0.207, high dose; 0.4 ± 0.1, *p* = 0.532, placebo group) (Figure 1).

Finally, we tried to ascertain whether plasma cytokines (IL-1β, IL-4, IL-6, IL-10, IL-15, INF-γ, and TNF-α), as well as soluble TNF-α and IL-6 receptors (sTNFR-I, sTNFR-II, sIL-6R) and C-reactive protein, were associated among them and with sensory variables, body mass index, free-fat mass, energy intake, quality-of-life score, as well as albumin and prealbumin. We did not find significant correlations between cytokines and sensory variables or dietary and nutritional status. However, plasma levels of C-reactive protein were positively associated with other inflammatory parameters (IL-6, TNF-α, and sTNFR-II). Additionally, IL-6 and IL-10 levels were negatively associated with plasma albumin.

## 3. Discussion

Based on previous preliminary experience of the working group, in the present study, we hypothesized that miraculin intake for about 3 months could improve the perception of bitter and metallic taste in patients with cancer and could increase their appetite for food, thereby improving their nutritional status and, as a consequence, improving their quality of life. In addition, the intervention with DMB was extended for 3 months, as this time is sufficient for the total pool of erythrocytes to be completely renewed [20]. Therefore, any change in the fatty acid profile derived from dietary changes would indicate an improved nutritional status.

In brief, our results suggest that unpleasant alterations in the perception of flavors enhance malnutrition, favoring the development of cachexia. DMB enhances flavor perception, which contributes to an improvement in the nutritional status of patients and, indirectly, alleviates the symptoms of cachexia.

The main finding of this study is that the regular consumption of a standard dose of DMB seems to ameliorate several biomarkers of inflammation and cachexia in malnourished patients with cancer undergoing antineoplastic treatment and suffering TDs.

After three months of treatment, patients tended to lose weight, BMI, and waist circumference. They significantly changed fat-free and total water. Higher doses of DMB had the greatest effect. Only patients taking DMB lost fat mass. The bioimpedance phase angle showed a greater improvement in patients consuming the standard dose of DMB than in those with a high dose or placebo. After three months, all patients regained the weight lost during the previous six months and improved their nutritional status. Two patients consuming a standard dose of DMB continued to show signs of malnutrition after the study ended [17].

Concerning the effects of the antineoplastic treatment, only plasma levels of the oncogene product sTNFR-II tended to decrease in patients who exhibited a reduction in tumor mass, regardless of DMB intervention.

Many cancer patients, particularly those with advanced disease, experience cancer cachexia, a complex and multifactorial syndrome thought to be the result of the actions of both host and tumor-derived factors and cytokines involved in a systemic inflammatory response to the tumor [16]. Cancer-associated malnutrition contributes to cachexia and can be associated with taste changes and food aversion [21,22,23]. Early intervention with oral nutritional supplementation has been reported to be effective in reversing malnutrition, leading to a positive impact on outcomes in some patients [16]. Hence, the benefits derived from the reduction in the inflammatory state observed in the present study in patients receiving the standard dose of DMB might be attributable to an improved nutritional status. This improvement is due to better taste perception and an increased dietary intake facilitated by DMB. Nevertheless, we were unable to detect significant correlations between plasma cytokines and sensory variables, energy intake, and nutritional status, which could be due to the relatively low number of patients included in this study and the high variance of those major outcome variables evaluated. DMB is a novel food rich in miraculin, a glycoprotein that activates sweet receptors in a pH-dependent manner, as miraculin does not possess a sweet taste on its own but relies on acidification of the oral cavity to elicit a change in taste perception [24]. This perception of the sour taste as sweet can persist for more than 1 h, although the intensity of the sweet taste decreases over time [25].

The efficacy and safety of DMB and miraculin treatment in cancer patients with taste disorders have been published by our group elsewhere [17]. We have reported that regular consumption of DMB improved energy intake, fat quantity and quality, fat-free mass, and quality of life in malnourished cancer patients receiving antineoplastic treatment [17]. Considering these results, a logical approach is to discern whether this improvement is reflected in the cachexia status. Indeed, improvements in cachexia parameters after nutritional interventions have already been reported [22,26]. For instance, the production of proinflammatory cytokines such as IL-6, IL-1β, and TNF-α is downregulated by the omega-3 PUFA eicosapentaenoic acid (EPA) in healthy people and cancer patients [22]. Arachidonic acid (AA) and omega-3 PUFAs are essential for cell signaling, cell structure, and membrane fluidity [27,28]. Both act as eicosanoid precursors, lipid-based signaling molecules that play a key role in innate immune responses [29]. AA and omega-3 PUFAs also produce a group of lipid-based pro-resolving mediators crucial for inhibiting proinflammatory signals: lipoxins, derived from arachidonic acid, and resolvins, protectins, and maresins, derived from omega-3 PUFAs. We have previously reported increased levels of selected PUFAs, including linoleic acid, AA, and omega-3 fatty acids (EPA and docosahexaenoic acid), following the habitual intake of a standard dose of DMB [17]. These findings could indicate a better food intake pattern, reflected in a better status of the PUFA profile. Remarkably, this improvement might be attributed not only to the antineoplastic treatment but also to supplementation with the miraculin-based food supplement DMB given that it was extended for three months, which is sufficient time for the complete renewal of the total pool of erythrocytes [20] and is in line with the amelioration observed in the biomarkers of inflammation. Furthermore, other authors reported that the effects of PIF, another cachectic biomarker, were also inhibited after EPA intake [30]. Other trials aimed to identify the physiological and clinical results of anti-cachexia treatment in patients with advanced cancer. MacCiò et al. treated gynecological cancer patients with megestrol acetate plus L-carnitine, a COX-2 inhibitor (celecoxib), and antioxidants versus only megestrol acetate alone; the combination treatment improved lean body mass, resting energy expenditure, fatigue, and quality of life [31]. TNF-α, IL-6, and INF-γ have been implicated as mediators of the metabolic changes associated with cancer cachexia [11,12]. The present study suggests decreased levels of INF-γ with the time course of intervention, mainly in patients who consumed DMB. Similar results were observed for the circulating levels of the soluble receptors sIL-6R and sTNFR-II. When receptors are bound by their respective cytokines, they initiate a series of intracellular signaling cascades. Dysregulation of the expression and secretion of these cytokines and their receptors is closely linked to the pathogenesis of inflammatory diseases and cancer [32]. In this sense, sIL-6R can stimulate various cellular responses, including proliferation, differentiation, and activation of inflammatory processes, which are key in regulating IL-6 responses. Elevated levels of sIL-6R have been documented in numerous clinical conditions, suggesting that its production is coordinated as part of a disease response [33]. On the other hand, TNF-α binds to two distinct receptors, TNFR-I and TNFR-II. TNFR-I has an intracellular death domain and induces inflammation, tissue degeneration, and programmed cell death. In contrast, TNFR-II lacks a death domain and mediates primarily homeostatic effects, including cell survival, proliferation, and tissue regeneration [32]. In some pathologic states, the production and release of sTNFRs may mediate the host response and determine the course and outcome of disease by interacting with TNF-α and competing with cell surface receptors [34]. In the tumor microenvironment, TNF-α via its receptors TNFR-I and TNFR-II plays a dual role in suppressing or promoting cancer proliferation and metastasis. TNFR-I can be expressed by nearly all cells, while TNFR-II can be highly expressed by tumor cells. In malignant cells, TNFR-II promotes tumor cell proliferation and is increasingly considered an oncogene because it is overexpressed in more than 20 types of cancer [35].

Concerning anti-inflammatory cytokines, the group that received the standard dose of DMB had the greatest increase in plasma IL-10. IL-10 is a double-edged sword in the immune system: it is a potent anti-inflammatory and immunosuppressive cytokine, but can also have immune-stimulatory properties [36].

**Figure 2:**
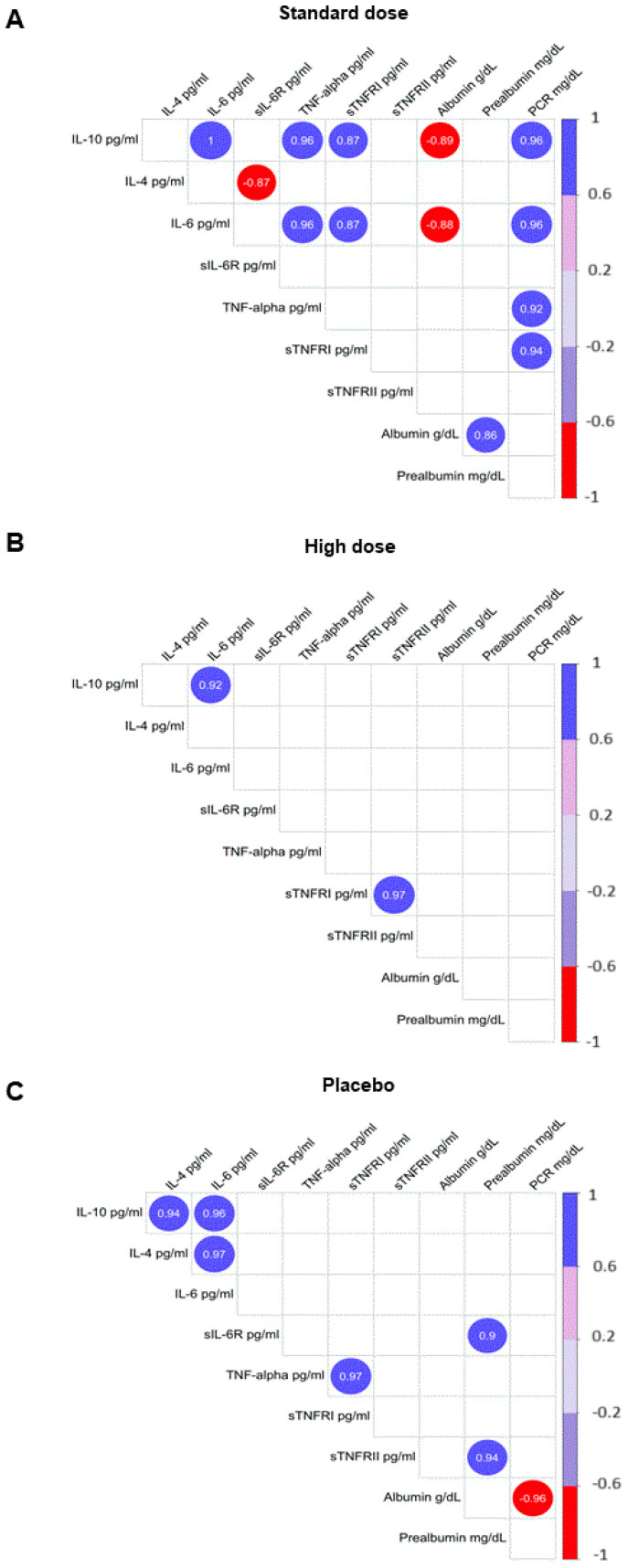
Correlations were observed between markers of inflammation **and selected** variables of nutritional status. (**A**) Standard dose of DMB; (**B**) high dose of DMB; (**C**) placebo. Plots show only significant and corrected associations. Red and purple lines indicate the correlation values, with negative correlations highlighted in red (−1) and positive correlations highlighted in purple (+1). IL, interleukin; PCR, C-reactive protein; TNFR, soluble TNF receptor; TNF-α, tumor necrosis factor-α. Only correlations with *p* > 0.05 are shown.

Data on inflammation in patients who received DMB (Figure 2) are within the range expected in patients who suffer from a state of inflammation. Based on these data, it could be hypothesized that there is a ‘correct functioning’ of the mechanisms involved in the course of the disease and the body’s responses, which could undoubtedly facilitate therapeutic approaches. Strikingly, these correlations were observed only in the group receiving the standard dose of DMB.

Another aspect of our results that deserves attention is the positive correlation between IL-6 and IL-10 in this group. This led us to question the balance of pro- and anti-inflammatory cytokines in patients. Several studies have highlighted the importance of this balance. For example, it has been reported that the ratio of IL-6 to IL-10 is strongly correlated with injury severity and the intensity of the anti-inflammatory response after trauma [37]. In addition, an imbalance in the systemic inflammatory response, marked by an increased IL-6/IL-10 ratio, is one of the possible factors contributing to the severity of primary open-angle glaucoma [38], gastric cancer [39], or COVID-19 patients [40]. Understanding the interplay between IL-6 and IL-10, which reflects the balance between proinflammatory and anti-inflammatory cytokines, is critical for identifying patients in a hyper-inflammatory state and will allow rational determination of the best treatment options for each patient [40]. One of the earliest events of cachexia is adipose tissue loss, often preceding skeletal muscle loss and predominantly driven by a combination of increased lipolysis and altered lipogenesis [9]. Therefore, we evaluated the effect of DMB intervention on the plasma levels of CNTF, a cytokine that induces the catabolism of stored fat, skeletal muscle protein, and liver glycogen [5]. CNTF and PIF are tumor-derived molecules that also induce protein degradation in skeletal muscle through the induction of the ubiquitin-proteasome proteolytic pathway [41]. Remarkably, both parameters were reduced only in the DMB standard-dose group, suggesting an amelioration of cachexia in this group of patients. In contrast to the standard dose, the use of a high dose of DMB caused patients to maintain an intensive sweet taste for a long time and, therefore, had a negative impact on dietary intake, which may explain why a dose greater than 150 mg is not as effective.

One of the major strengths of the present pilot study is that it was carefully designed as a randomized, triple-blind, placebo-controlled intervention trial. In our view, although the number of patients included did not allow us to detect major differences in the evaluated parameters, it is undeniable that patients treated with the DMB supplement showed an improvement in their cachectic state in general and their immune profile in particular compared with those in the placebo group, especially at the standard dose of 150 mg (Table 2). Consequently, this effect should not be exclusively attributed to the antineoplastic treatment alone but rather to the intervention with DMB. Thus, the success in the antineoplastic treatment was not correlated to any of the cytokines, receptors, and tumor factors determined, except for sTNFR-II, which is a well-known protein highly expressed in tumor cells [42]. The major limitation of the present study is the number of subjects, although the relevance and solidity of the results are of great importance and cannot be the result of chance alone. Other studies have reported improvements in cachexia and inflammation in cancer patients after nutritional interventions, especially with long-chain omega-3 PUFAs [22,43]. However, improving sensory alterations in cancer patients and promoting a better dining experience, which leads to an improvement in nutritional status and thus a decrease in biochemical parameters of inflammation and malnutrition, is a novel approach that has not previously been implemented.

This improvement also means an increase in the quality of life of patients and, as a result, a better ability to cope with harsh treatment and side effects [17]. We are aware that systemic treatment of cancer patients has a key and unique role in the amelioration of the disease. However, as all patients in our study received an antineoplastic treatment, particularly chemotherapy, the intake of DMB should be considered responsible for at least part of the observed benefits. DMB does not seem to have a direct role in tumorigenesis; however, its indirect role in improving the nutritional status and particularly increasing the essential and long-chain PUFA status could explain its effects on inflammation and cachexia. However, the dried miracle berries are rich in several bioactive compounds, mainly polyphenols, triterpenoids, and amides, and we cannot exclude a direct effect of DMB on cancer progression; in this sense, DMB has been shown to exhibit antitumoral activities in vitro [44,45], although studies in humans are lacking.

Regarding the use of different doses of DMB, in this study, we aimed to elucidate whether the intake of this product followed a dose/effect relationship in patients. For the choice of the dose of this *novel food* evaluated in the present work, we based ourselves on the European Union recommended dose for healthy individuals (0.6 g/d) [17]. We chose a slightly lower dose than the maximum recommended for healthy people for the ‘standard dose’ group. In contrast, the ‘high dose’ group received twice this amount. Our rationale was that since these patients have a less acute perception or sense of taste, a higher dose could compensate for this deficiency. However, we found worse results in that group. This result could be because the sweet taste lingers in the mouth for more than 30 min, which could be classified as an undesirable effect and therefore a lower enjoyment of meals by the patients.

Since nutritional therapy is key in the treatment of cancer cachexia, we still need to clarify the effects of nutrients and food supplements on the metabolic mechanisms and pathways affected by this disorder. This aspect is key, keeping in mind that in advanced cancer, optimal nutrition is required to help patients tolerate aggressive or long-term treatments, maintain quality of life, and cope with disease progression. As a consequence, Studies on the molecular signaling pathway and specific mechanisms of action of bioactive compounds within food supplements could allow their use to target the main pathogenic mechanisms of cancer cachexia and malnutrition.

## 4. Materials and Methods

### 4.1. Study Design and Patients

The pilot CLINMIR study was designed to evaluate the efficacy and safety of the DMB food supplement based on sensory function, nutritional status, dietary intake, quality of life, and the fatty acid profile of erythrocytes in adult malnourished cancer patients with TDs undergoing active antineoplastic therapy [46], and the complete protocol as well as the results of the major characteristics of patients and main outcomes, along with the DMB composition, have been reported elsewhere [17,46]. The study was conducted following the Declaration of Helsinki. The clinical trial protocol was approved by the Scientific Research and Ethics Committee of the Hospital Universitario La Paz (HULP), Madrid (Spain) in version 1 in June 2022 and protocolled by the HULP Code 6164. Informed consent was obtained from all subjects involved in the study. This clinical trial was registered at http://clinicaltrials.gov (accessed on 2 June 2024) (Clinical Trial NCT05486260). Briefly, a triple-blind, randomized, placebo-controlled intervention clinical trial with three arms was conducted. Thirty-one malnourished patients with cancer of various etiologies receiving systemic therapy and presenting TDs were included. Malnutrition diagnosis was assessed using the Global Leadership Initiative on Malnutrition (GLIM) criteria and morpho-functional assessment of disease-related malnutrition [20]. Sensory disturbances were assessed by electrogustometry [17]. Cancer patients did not suffer from any other additional disease. Detailed inclusion and exclusion criteria have previously been reported [17,46].

The inclusion criteria included adult patients with cancer and systemic antineoplastic treatment for at least three months who had a weight loss of ≥5%. All of them were capable of oral intake of food and drinks. The characteristics of enrolled patients and the grade of disease have been reported elsewhere [47] and are summarized in Table S1. The exclusion criteria were patients with cancer participating in another clinical trial, with enteral or parenteral nutrition, major gastrointestinal, metabolic, neurological, and mental diseases, eating disorders, and severe digestive toxicity due to treatment with chemo-radiotherapy, as well as the willingness to consume the DMB food supplement. The patients were randomly assigned to three groups and received one tablet of 150 mg of DMB (standard dose, *n* = 10), 300 mg of DMB (high dose, *n* = 11), or a placebo (*n* = 10) for three months before each main meal.

Anthropometric measures were taken, and the patients underwent electrical bioimpedance testing and a Sniffin’ Sticks smell test. The research team took the opportunity to explain the healthy eating and physical exercise guidelines for cancer patients (baseline) [17,46].

After three months, the patients underwent tests to assess their physical and sensory abilities. The patients’ food diaries and completed quality-of-life questionnaires were collected, as well as product efficacy questionnaires, product consumption control, tolerance records, and adverse effects records. The patients were encouraged to reinforce their nutritional treatment and physical activity. A blood sample was taken to determine biochemical parameters and fatty acids in their erythrocytes.

For some of the parameters evaluated, data were obtained at baseline, mid-time, and the end of the intervention. However, the results reported in this article were obtained from data only at the beginning and end of the study for cytokines and tumor growth factors.

Specialized nutritionists and endocrinologists at the University Hospital La Paz, Madrid, Spain, performed measurements on the same patients. A specialized oncologist assessed the progression of cancer using computer-assisted tomography.

Throughout the patient interviews with nurses during their treatment, the patients were given various nutritional tips. The nurses also provided healthy recipes and encouraged them to follow a balanced diet. All of this is reflected in López-Plaza et al., 2023, which details the complete study protocol [46].

### 4.2. Blood Samples

Blood samples were collected in the morning (approx. 8:00 a.m.) by trained personnel at the University Hospital La Paz Extraction Unit (Madrid, Spain), coinciding with blood tests before chemotherapy to avoid more punctures and hospital visits than necessary. Blood samples were obtained at baseline, mid-time, and the end of the intervention in tubes containing EDTA and were centrifuged immediately at 1000× *g* for 10 min. Plasma was isolated and stored at −80 °C until analysis [17,46].

### 4.3. Determination of Plasma Cytokines, Tumor Cachexia Factors, and Biochemical Parameters

Based on previous reports [48,49,50,51], relevant molecules previously described to be associated with inflammatory processes and cancer cachexia were selected for plasma analysis. Specifically, TNF-α, IL-6, IL-1β, IL-4, IL-10, IFN-γ, IL-15, soluble IL-6 receptor (sIL-6R), soluble TNF receptor type I (sTNFR-I), and soluble TNF receptor type II (sTNFR-II) were analyzed by the X-MAP Luminex multiplex enzymoimmunoassay platform using specific antibodies as previously described [52]. Human CNTF and PIF were analyzed by enzyme-linked immunosorbent assay (ELISA) following the kit instructions provided by the manufacturers. Specific bead panels and ELISA kits used with their corresponding variation coefficients are detailed in Table 2. The Biochemistry Laboratory at Hospital La Paz, an ISO-certified laboratory, performed biochemical analyses using an Olympus AU5400 Automated Chemistry Analyzer (Olympus Corporation, Izasa, CA, USA). A summary of key biochemical parameters is provided in Table S2.

The nutritional status of the subjects was evaluated following the GLIM criteria, alongside the measurement of electrical (electrogustometry) and chemical taste perception (taste strips) While cachexia is a severe form of malnutrition, the present study also included patients with moderate malnutrition over 18 years of age with cancer and antineoplastic treatment (chemotherapy and any other treatment such as radiotherapy, immunotherapy, etc., for at least three months) who had a weight loss of ≥5%.

Bioelectrical impedance analysis (BIA) was used to assess muscle mass. Symptoms were considered to evaluate food assimilation and primary infections. Trauma/acute conditions were associated with inflammation. Malnutrition was classified as moderate or severe. Nutritional status was assessed at all visits [17,46].

### 4.4. Taste Threshold

Electrogustometry was used to evaluate taste thresholds. Electrical taste testing is an excellent method for quantifying human taste objectively [53]. If a patient with cancer and taste distortion consumes miraculin-based food supplements, their taste threshold is expected to be improved by reducing their taste perception threshold (measured in decibels, dB) via electrical stimulation at baseline, one month following DMB intervention, and three months thereafter, as measured by electrical stimulation [17,46].

The results related to the evaluation of taste disorders and their evolution, including those measured by electrogustometry, have been reported by our group elsewhere [17] and in Table S3.

### 4.5. Statistical Analysis

The results are expressed as the mean + standard error of the mean. To determine whether there is a significant difference between the treatment and the reference value and whether there is a significant interaction between time and treatment, a general linear mixed model of covariance (ANCOVA) was employed to evaluate each of the cytokine variables, using cancer treatment success as a covariate expressed as a dichotomous variable [54]. To conduct a robust analysis, the linear mixed model was carried out using the lme4 package in the R programming language (R Project for Statistical Computing https://www.r-project.org/ (accessed on 2 June 2024) [55]. Since the lme4 package employs modern, efficient linear algebra methods, such as those implemented in the Eigen package, and reference classes to prevent unnecessary copying of large objects, it may be faster and more memory-efficient than other programs. Moreover, the software is capable of developing generalized linear mixed models using the glmer function, which maximizes the amount of information that can be obtained when lost patients are included in some of the analyzed conditions [54]. In brief, the linear mixed model with the lme4 package was fitted using the restricted maximum likelihood approach, which is a particular form of maximum likelihood estimation that does not assume a maximum likelihood fit of all the information. Instead, it uses a likelihood function calculated from a transformed set of data, so nuisance parameters have no effect. The Welch–Satterthwaite method was used to calculate an approximation to the effective degrees of freedom of a linear combination of independent sample variances, which is also referred to as the pooled degrees of freedom. The robust ANCOVA avoids the constraints of homogeneity of regression slopes and other distributional assumptions. These tests compare trimmed means at different points along the covariate.

The Shapiro–Wilk test was used to test the assumptions of normality. Additionally, based on Pearson’s correlations, we examined the relationships between inflammatory variables in plasma and taste acuity, body mass index, free-fat mass, energy intake, quality-of-life score, as well as albumin and pre-albumin in patients with cancer. Studio’s corrplot function [56] was used to express associations by correcting multiple tests with the false discovery rate procedure [57]. Plots show only significant and corrected associations. Red and purple lines indicate the correlation values, with negative correlations highlighted in red (−1) and positive correlations highlighted in purple (+1) (Figure 2). All results were analyzed using the R Project for Statistical Computing (https://www.r-project.org/) [55], and a *p*-value < 0.05 was considered to indicate statistical significance.

## 5. Conclusions

In conclusion, regular consumption of a standard dose of the food supplement DMB containing miraculin along with a systemic antineoplastic treatment could contribute to improving biomarkers of inflammation and cachexia in malnourished patients with cancer and TDs. Future studies with a larger sample size and with selected types of cancer are warranted to corroborate and extend new findings in this field.

## Figures and Tables

**Table 1:** Plasma levels of several biomarkers of inflammation and cachexia in malnourished cancer patients after three months of intervention with DMB or placebo.

Variables	Standard-Dose DMB *n* = 10		High-Dose DMB *n* = 11		Placebo *n* = 10		*p*-Value		
	Baseline	3 Months	Baseline	3 Months	Baseline	3 Months	Time (T)	Treatment (t)	T × t
	Cytokines (pg/mL)								
IL-6	4.7 ± 1.5	3.9 ± 2.1	5.0 ± 1.1	3.3 ± 1.4	5.9 ± 3.9	3.8 ± 2.0	0.142	0.896	0.900
IL-1β	5.6 ± 1.6	4.7 ± 0.8	4.3 ± 0.5	4.1 ± 1.3	4.5 ± 0.7	3.4 ± 0.3 *	*0.093*	0.838	0.493
TNF-α	16.5 ± 2.2	16.4 ± 4.0	14.2 ± 2.5	9.3 ± 1.3	14.1 ± 1.6	12.6 ± 1.9	0.316	0.220	0.648
INF-γ	31.4 ± 8.9	25.3 ± 7.2 *	18.1 ± 2.1	18.8 ± 4.7	20.0 ± 4.3	18.5 ± 3.5	0.229	0.530	0.206
IL-15	11.7 ± 4.0	24.9 ± 13.5	42.1 ± 19.6	17.8 ± 7.4	10.4 ± 3.8	9.4 ± 1.1	0.555	0.233	0.347
IL-4	36.9 ± 11.3	27.3 ± 4.9	38.2 ± 13.4	37.3 ± 19.7	71.1 ± 40.9	48.5 ± 24.6	**0.042**	0.499	0.379
IL-10	9.3 ± 1.9	28.1 ± 18.8	12.8 ± 3.7	15.9 ± 6.6	17.1 ± 13.4	13.1 ± 6.6	0.386	0.826	0.454
CNTF	358.8 ± 122.7	300.0 ± 109.0	332.9 ± 115.5	413.3 ± 186.6	339.9 ± 169.6	378.3 ± 134.0	0.756	0.966	0.911
	Soluble receptors (μg/mL)								
sIL-6R	11.0 ± 2.6	10.2 ± 1.7	9.7 ± 1.4	9.3 ± 2.7	8.6 ± 1.6	8.2 ± 2.0	0.624	0.530	0.979
sTNFR-I	1.3 ± 0.3	1.3 ± 0.3	1.2 ± 0.3	0.9 ± 0.2 *	0.9 ± 0.1	1.2 ± 0.2	0.080	0.827	0.508
sTNFR-II	7.5 ± 1.9	4.6 ± 0.7 *	6.8 ± 1.5	4.8 ± 1.1	5.1 ± 0.9	5.8 ± 1.6	**0.011**	0.615	0.279
	Tumor-derived factors (μg/mL)								
PIF	14.8 ± 2.3	11.7 ± 2.3 *	15.2 ± 2.3	10.6 ± 1.6	10.5 ± 0.3	10.0 ± 1.1	**0.021**	0.464	0.323

Abbreviations: CNTF, ciliary neurotrophic factor; IFN, interferon; IL, interleukin; PIF, proteolysis-inducing factor/dermcidin; sIL-6R, soluble IL-6 receptor; sTNFR, soluble TNF receptor; TNF-α, tumor necrosis factor-alpha. A general linear mixed model of covariance (GLM-ANCOVA) was performed using cancer treatment success as a covariate. * *p* < 0.05, baseline vs. 3 months for each treatment. The bolded values represent significance, and the italicized values represent significance levels between 0.05 and 0.1.

**Table 2:** Bead panels for enzyme-linked immunosorbent assay, analytes, and variation coefficients for cytokines and cachexia tumor factors analyzed.

Bead Panels or Enzyme-Linked Immunosorbent Assay	Analyte	Assay CV
HSTCMAG-28SK-06 (EMD Millipore Corporation, St. Louis, MO, USA)	TNF-α	12.64
	IL-6	19.31
	IL-1β	7.86
	IFN-γ	7.14
	IL-4	16.89
	IL-10	8.87
HCYTA-60K (EMD Millipore Corporation, St. Louis, MO, USA)	IL-15	9.35
HSCRMAG-32K-03 (EMD Millipore Corporation, St. Louis, MO, USA)	sIL-6R	8.83
	sTNFR-I	3.76
	sTNFR-II	5.65
CSB-E04527h (Cusabio, Wuhan, China)	CNTF	5.97
CSB-E13626h (Cusabio, Wuhan, China)	PIF	8.54

Abbreviations: CNTF, ciliary neurotrophic factor; IFN, interferon; IL, interleukin; PIF, proteolysis-inducing factor/dermcidin; sIL-6R, soluble IL-6 receptor; sTNFR, soluble TNF receptor; TNF, tumor necrosis factor.

## Data Availability

The corresponding author should be contacted reasonably to request access to the datasets used and/or analyzed in the current study. The data are not publicly available due to ethical reasons.

## Supplementary Materials

The following supporting information can be downloaded at: https://www.mdpi.com/article/10.3390/ph18050622/s1, Table S1. Cancer types and chemotherapy characteristics of patients included in the CLINMIR study. Table S2. Selected anthropometric, physiological, quality of life and biochemistry parameters. Table S3. Electrical taste perception depending on treatment.
