# Supplementary material for: Miraculin Can Contribute to a Reduction in Inflammatory Biomarkers and Cachexia in Malnourished Patients with Cancer and Taste Disorders
Source: Pharmaceuticals (Basel). 2025 Apr 25;18(5):622. doi: 10.3390/ph18050622 (PMC12115146; doi:10.3390/ph18050622)
Supplement: Supplementary file 1 [file pharmaceuticals-18-00622-s001.zip › pharmaceuticals-3583878-supplementary.pdf]

Table S1, previously reported by us in the article entitled ¨Effect of a Novel Food Rich in Miraculin on the Intestinal Microbiome of Malnourished Patients with Cancer and Dysgeusia¨summarizes the cancer types as well as the radiotherapy and chemotherapy received by the patients. We did not find significant difference in distribution among the groups of those variables.

Table S1. Cancer types and chemotherapy characteristics of patients included in the CLINMIR study

Variables		DMB 150 mg	DMB300mg	Placebo	p-value
Sex	Female(%)	70	45.5	60	0.517
	Male(%)	30	54.5	40	
Age	years	59.9 ± 15.1	58.9 ± 4.9	61.3 ± 11.2	0.891
Weight	kg	61.4 ± 11.1	62.0 ± 14.1	62.6 ± 10.7	0.941
Weight lost in last 6 months	%	7.5 ± 6.0	8.7 ± 7.1	7.2 ± 8.0	0.868
BMI	kg/m^2	21.9 ± 3.6	22.0 ± 3.3	22.9 ± 3.4	0.737
Type of cancer					
Head and neck	%	0.00	9.10	0.00	
Colorectal	%	30.00	27.30	20.00	
Esophagus	%	10.00	0.00	10.00	
Stomach	%	0.00	9.10	10.00	
Liver	%	0.00	9.10	10.00	
Breast	%	10.00	18.20	10.00	0.895
Neuroendocrine	%	10.00	0.00	0.00	
Ovary	%	10.00	18.20	0.00	
Pancreas	%	10.00	9.10	10.00	
Lung	%	10.00	0.00	10.00	
Others	%	10.00	0.00	20.00	
Chemotherapy	%	100.0	100.0	100.0	1
Radiotherapy	%	20.00	12.50	0.00	0.594
Smoking status (yes)	%	30.00	27.30	40.00	0.605
Alcohol use (yes)	%	20.00	27.30	10.00	0.393
Dental Disease (yes)	%	0.00	0.00	0.00	1

BMI, body mass index. Values are expressed as mean± standard deviation

Taken from Plaza-Diaz J, Brandimonte-Hernández M, López-Plaza B, Ruiz-Ojeda FJ, Álvarez-Mercado AI, Arcos-Castellanos L, Feliú-Batlle J, Hummel T, Palma-Milla S, Gil A. Effect of a Novel Food Rich in Miraculin on the Intestinal Microbiome of Malnourished Patients with Cancer and Dysgeusia. Nutrients. 2025 Jan 10;17(2):246. doi: 10.3390/nu17020246. PMID: 39861376; PMCID: PMC11767858.

Table S2. Selected anthropometric, physiological, quality of life and biochemistry parameters.

Variables	Standard-dose DMB n=10		High-dose DMB n=11		Placebo n=10		p-value		
	Baseline	3 months	Baseline	3 months	Baseline	3 months	Time (T)	Treatment (t)	T x t
Anthropometric									
Body mass index (kg/m2)	21.9 ± 1.1	20.9 ± 13	22.0 ± 1.0	22.7 ± 1.4	22.9 ± 1_1	23.9 ± 1.1	0.587	0.671	0.313
Free-fat mass (kg)	16.6 ± 0.7	16.7 ± 0.8	16.8 ± 03	16.4 ± 0.5	16.6 ± 03	17.2 ± 0.2	0.675	0.953	0.077
Physiological									
Taste threshold (dB}	18.9 ± 3.8	10.3 ± 4.0*	19.4 ± 4.0	18.1 ± 7.6	20,2 ± 3.8	13.3 ± 5.1	**0.037**	0.747	0.618
Quality of life									
Quality of life score (percentage)	66.7 ± 3.9	69.8 ± 5.6	46.2 ± 4.8	40.3 ± 3.3	57.4 ± 7.8	78.6 ± 3.6	0.126	**0.002**	**0.023**
Biochemistry									
Albumin (g/dL)	43 ± 0.05	4.1 ± 0.2	4.2 ± 0.09	43 ±111	43 ±111	43 ± 0.08	0.292	0.661	0.196
Prealbumin (mg/dL)	23.8 ± 2.2	20.3 ± 2.0	22.1 ± 2.4	24.9 ± 4.7	19.9 ± 1.8	20.6 ± 1.7	0.915	0.674	0.389

Values are expressed as mean f SEM. General Linear Mixed Model of Covariance (GLM ANCOVA) was performed using cancer treatment success as a covariate. *p<0.05, baseline vs 3 months for each treatment. The bolded values represent significance.

Table S3. Electrogustometry test results

Taste perception was evaluated using electrogustometry. To quantify human taste perception objectively, electrical taste testing is an excellent method. Functional imaging studies have shown that lingual electrical stimulation activates the same brain regions as chemical stimulation. Patients with cancer and taste distortion and who consume miraculin-based food supplements are expected to improve their taste perception by reducing their taste perception threshold (measured in decibels, dB) via electrical stimulation at baseline, one month after the intervention with DMB, and three months thereafter, as measured by electrical stimulation. An electrogustometer (SI-03 Model, Sensonics International, Haddon Heights, NJ, USA) was used to measure the threshold for an electrically induced taste stimulus. An electrode is placed on the tongue to apply the electric stimulus. To familiarize the patient with the electrical stimulus, a first stimulus (30 dB) is administered. Upon determining the threshold, stimulation begins at the zero-stimulus amplitude and increases progressively until the patient identifies the stimulus. A stimulus-response staircase and the two-down one-up forced-choice single staircase were used to measure detection thresholds (López-Plaza et al. 2024).

Table S3. Electrical taste perception depending on treatment.

						p-Value		
			**Standard Dose of DMB**	**High Dose of DMB**	**Placebo**	**Time (t)**	**Treatment (T)**	**T × t**
Right side	(dB)	Baseline	17.7 ± 13.2	19.3 ± 14.0	17.9 ± 13.4	0.200	0.393	0.499
		1 week	18.5 ± 10.4	14.5 ± 15.5	15.2 ± 13.5			
		1 month	17.9 ± 16.3	20.0 ± 15.4	16.5 ± 17.3			
		2 months	6.9 ± 10.8	20.8 ± 14.1	10.8 ± 11.9			
		3 months	10.9 ± 11.1	18.0 ± 18.8	16.7 ± 17.1			
Left side	(dB)	Baseline	20.0 ± 12.5	19.7 ± 14.0	22.6 ± 13.8	0.444	0.544	0.946
		1 week	15.9 ± 12.9	19.1 ± 16.0	17.1 ± 15.8			
		1 month	12.1 ± 15.3	17.7 ± 15.1	14.7 ± 15.4			
		2 months	9.6 ± 13.5	18.4 ± 16.2	18.4 ± 13.1			
		3 months	9.8 ± 13.5	18.3 ± 18.4	9.9 ± 12.5			

Values are expressed as mean ± standard deviation

Taken from López-Plaza B, Álvarez-Mercado AI, Arcos-Castellanos L, Plaza-Diaz J, Ruiz-Ojeda FJ, Brandimonte-Hernández M, Feliú-Batlle J, Hummel T, Gil Á, Palma-Milla S. Efficacy and Safety of Habitual Consumption of a Food Supplement Containing Miraculin in Malnourished Cancer Patients: The CLINMIR Pilot Study. Nutrients. 2024 Jun 17;16(12):1905. doi: 10.3390/nu16121905. PMID: 38931260; PMCID: PMC11207068.
